# Compliance with Valproate Pregnancy Prevention Programme Documentation in Women of Reproductive Age: A Clinical Audit From a Tertiary Mental Health Service in the UAE

**DOI:** 10.1192/bjo.2026.11690

**Published:** 2026-06-30

**Authors:** Amjad Ibrahim Almasri, Saman Ahmed, Syed Fahad Javaid

**Affiliations:** 1 Behavioural Sciences Institute, Al Ain, UAE; 2 Department of Psychiatry, College of Medicine and Health Sciences, United Arab Emirates University, Al Ain, UAE

## Abstract

**Aims:**

Valproic acid is an effective mood stabiliser but carries significant teratogenic risks when prescribed to women of reproductive age. Clinical guidance recommends that women are fully informed of these risks, advised on effective contraception, and that discussions are clearly documented as part of shared decision-making. This audit aimed to assess the extent and quality of documentation of counselling on teratogenic risks, pregnancy prevention, and informed consent in women of reproductive age prescribed valproic acid at a tertiary mental health service in the United Arab Emirates (UAE).

**Methods:**

A retrospective clinical audit was conducted at the Behavioural Sciences Unit, Al Ain Hospital. Pharmacy records and electronic medical notes were reviewed for all female patients aged 18–50 years who were prescribed valproic acid during the audit period. Patients with intellectual disability, cerebral palsy, or severe cognitive impairment were excluded because of limitations in informed consent. Audit standards were derived from the National Institute for Health and Care Excellence (NICE) and Medicines and Healthcare products Regulatory Agency (MHRA) guidance on the Valproate Pregnancy Prevention Programme, focusing on documenting counselling about teratogenic risks, contraception, pregnancy prevention, and informed consent. Data were analysed descriptively as frequencies and percentages.

**Results:**

A total of 56 female patients of reproductive age were prescribed valproic acid during the audit period. Seven patients were excluded, leaving 49 for analysis. Documented counselling regarding the teratogenic risks of valproic acid, including congenital malformations and neurodevelopmental impairment, was present in 12 patients (24.5%). In these cases, documentation included discussion of pregnancy prevention and contraceptive advice. The remaining 37 patients (75.5%) had either partial documentation or no documented evidence of counselling regarding teratogenic risks, contraception, or pregnancy prevention. Documentation of partner involvement in discussions was noted in 5 cases (10.2%). Only 8 patients (16.3%) had a discussion of alternative treatment options recorded in the medical notes.

**Conclusion:**

This audit found major gaps in documenting counselling and consent about valproic acid’s risks in women of reproductive age. While counselling may happen, poor documentation poses medicolegal and safety concerns. Interventions like electronic prompts, templates, multilingual leaflets, and clinician awareness are needed to boost guidance compliance. A follow-up is advised to evaluate these actions and ensure safer prescribing.

No financial sponsorship has been received for this project.

